# Predicting on-going hemorrhage and transfusion requirement after severe trauma: a validation of six scoring systems and algorithms on the TraumaRegister DGU^®^

**DOI:** 10.1186/cc11432

**Published:** 2012-07-20

**Authors:** Thomas Brockamp, Ulrike Nienaber, Manuel Mutschler, Arasch Wafaisade, Sigune Peiniger, Rolf Lefering, Bertil Bouillon, Marc Maegele

**Affiliations:** 1 Institute for Research in Operative Medicine (IFOM), University of Witten/Herdecke, Cologne-Merheim Medical Center (CMMC), Ostmerheimer Str. 200, D-51109 Cologne, Germany; 2 Department of Traumatology and Orthopedic Surgery, Cologne-Merheim Medical Center (CMMC), University of Witten/Herdecke, Ostmerheimer Str. 200, D-51109 Cologne, Germany; 3 Academy for Trauma Surgery, Luisenstr. 58/59, D-10117 Berlin, Germany

## Abstract

**Introduction:**

The early aggressive management of the acute coagulopathy of trauma may improve survival in the trauma population. However, the timely identification of lethal exsanguination remains challenging. This study validated six scoring systems and algorithms to stratify patients for the risk of massive transfusion (MT) at a very early stage after trauma on one single dataset of severely injured patients derived from the TR-DGU (TraumaRegister DGU^® ^of the German Trauma Society (DGU)) database.

**Methods:**

Retrospective internal and external validation of six scoring systems and algorithms (four civilian and two military systems) to predict the risk of massive transfusion at a very early stage after trauma on one single dataset of severely injured patients derived from the TraumaRegister DGU^® ^database (2002-2010). Scoring systems and algorithms assessed were: TASH (Trauma-Associated Severe Hemorrhage) score, PWH (Prince of Wales Hospital/Rainer) score, Vandromme score, ABC (Assessment of Blood Consumption/Nunez) score, Schreiber score and Larsen score. Data from 56,573 patients were screened to extract one complete dataset matching all variables needed to calculate all systems assessed in this study. Scores were applied and area-under-the-receiver-operating-characteristic curves (AUCs) were calculated. From the AUC curves the cut-off with the best relation of sensitivity-to-specificity was used to recalculate sensitivity, specificity, positive predictive values (PPV), and negative predictive values (NPV).

**Results:**

A total of 5,147 patients with blunt trauma (95%) was extracted from the TR-DGU. The mean age of patients was 45.7 ± 19.3 years with a mean ISS of 24.3 ± 13.2. The overall MT rate was 5.6% (*n *= 289). 95% (*n *= 4,889) patients had sustained a blunt trauma. The TASH score had the highest overall accuracy as reflected by an AUC of 0.889 followed by the PWH-Score (0.860). At the defined cut-off values for each score the highest sensitivity was observed for the Schreiber score (85.8%) but also the lowest specificity (61.7%). The TASH score at a cut-off ≥ 8.5 showed a sensitivity of 84.4% and also a high specificity (78.4%). The PWH score had a lower sensitivity (80.6%) with comparable specificity. The Larson score showed the lowest sensitivity (70.9%) at a specificity of 80.4%.

**Conclusions:**

Weighted and more sophisticated systems such as TASH and PWH scores including higher numbers of variables perform superior over simple non-weighted models. Prospective validations are needed to improve the development process and use of scoring systems in the future.

## Introduction

Trauma is the leading cause of death world-wide in persons under the age of 40 years and accounts for approximately 10% of all deaths in general [1,2]. Almost 50% of patients with lethal outcome within the first 48 hours after trauma die from uncontrolled exsanguinating hemorrhage [2-4]. On average, one in four trauma patients arriving in the trauma bay is already coagulopathic upon admission and about one third of transfused patients require massive transfusion [5,6]. Meanwhile, it has been shown that the early activation of massive transfusion (MT) protocols including balanced ratios of blood products and the early substitution of coagulation factors may improve survival [7-9]. However, the timely identification of patients in need for aggressive hemostatic resuscitation remains challenging.

Over the past years, a lot of effort has been put into identifying parameters to analyze and measure pathophysiological pathways occurring in the setting of the acutely bleeding trauma patient and to mirror the coagulation state. However, parameters like the international normalized ratio (INR) or the activated partial thromboplastin time (aPPT) by themselves have been assessed with only bounded significance [10,11]. Recent publications have shown that ROTEM^® ^testing might be superior to analyze coagulopathic states in the early phase after trauma [12-14]. On the clinical level, several scoring systems and algorithms have been suggested for the early stratification of trauma patients at risk for massive transfusion. These systems have been developed on the basis of retrospective datasets from trauma patients derived from different trauma databases and registries. Usually developed and validated on one registry only using a split-data approach, their validity when subjected to external validation has only been assessed to a limited extent.

Several scoring systems and algorithms to predict the risk for MT have been introduced [15-22]. In principle, these systems have been developed and validated using retrospective datasets from civilian and combat trauma patients. In the present work, our aim was to compare six frequently used scoring systems and algorithms with the potential to early identify trauma patients at risk for massive transfusion and to validate all six scores on one dataset, including severely injured trauma patients derived from the TraumaRegister DGU^® ^(TR-DGU, Trauma registry of the German Trauma Society).

## Materials and methods

In the present study, we queried MEDLINE (1969-July 2011) and The Medical Algorithms Project for scoring systems and algorithms with the potential to identify patients at risk for MT at a very early stage after trauma [23]. Only scores including parameters that could be reproduced by the data captured in the TraumaRegister DGU^® ^were considered. MT was defined by administration of ≥ 10 units of packed red blood cells (pRBC) between arrival at the emergency room (ER) and the intensive care unit (ICU). Patients who had received hemostatic agents such as fibrinogen, prothrombin complex concentrate (PCC), recombinant activated factor VII (rFVIIa) or any antifibrinolytics with potential influence on the amount of administered pRBCs were excluded from the study. Furthermore, the TraumaRegister DGU^® ^captures information on additional hemostatics dichotomously, without capturing information about the amount or the time of administration.

### TraumaRegister DGU^®^

The TraumaRegister DGU^® ^is a prospective multi-center database with standardized documentation of patients suffering from severe trauma and thus requiring admission to an intensive care unit [24]. Preclinical death, burns or poisonings and femur neck fractures in elderly people are not included. This registry comprises detailed information on demographics, clinical and laboratory data, as well as a variety of standardized scoring systems on injury severity, for example, the Glasgow coma score [25], the injury severity score (ISS) [26], the abbreviated injury scale (AIS) [26], and the trauma and injury severity score (TRISS) [27]. Interventions are documented according to the International Classification of Procedures in Medicine. The TraumaRegister DGU^® ^is approved by the review board of the German Society for Trauma Surgery and is in compliance with the institutional requirements of its members. From 2002 to 2010, a total of 56,573 patients from 407 participating hospitals from seven different countries were included. Data are handled anonymously, and case identification is possible only through the participating hospital [22]. The TraumaRegister DGU^® ^is a voluntary registry, and participation is free of charge. The trauma registry is approved by the review board of the German Trauma Society (DGU) and is in compliance with the institutional requirements. As the TraumaRegister DGU^® ^is an anonymous registry, the Institutional Review Board waived the need for informed consent.

### Dataset

Data from 56,573 patients entered into the registry between 2002 and 2010 were screened to extract one complete dataset matching all variables needed to calculate all scoring systems and algorithms assessed in this study. We only used data for primary-admitted patients, ≥ 18 years of age, where the amount of administered units of pRBC was known. Moreover, only patients who had survived until ICU admission were considered, to avoid bias from early deaths prior to administration of any blood product or MT.

### Massive transfusion prediction scores

Our literature search identified six scoring systems and algorithms with the potential to identify patients at risk for MT at a very early stage after trauma, and that could be rebuild by our selected dataset (Table 1). The following scoring systems and algorithms were assessed: trauma-associated severe hemorrhage score (TASH) score, Prince of Wales Hospital/Rainer score (PWH), Vandromme score, assessment of blood consumption (ABC) score, Schreiber score and Larson score. Four scores were derived from civilian datasets and two from combat databases. While the TASH score has initially been developed and validated on the TraumaRegister DGU^® ^database, this score was again internally revalidated using a different patient cohort. In contrast, all other five scores and algorithms have been developed and validated on other databases and were thus externally validated by the present study.

**Table 1:** Parameters of compared scores.

Score Civilian or Military Database Number of patients	**TASH **[22,28] Civilian 4527		**Rainer (PWH) **[19] Civilian 1891		**Vandromme **[21] Civilian 514		**ABC **[18] Civilian 596		**Schreiber **[20] Military 558		**Larson **[29] Military 1124	
**Variable**	**Value**	**Pts**	**Value**	**Pts**	**Value**	**Pts**	**Value**	**Pts**	**Value**	**Pts**	**Value**	**Pts**

Gender	male	1										
Pelvic fracture (AIS 5 ≥ 5)	clinically unstable	6	displaced (AIS 5 ≥ 4)	1								
Femur fracture (AIS 5 ≥ 3)	open and/or dislocated	3										
Free IF (FAST) (AIS 4 ≥ 3)	present	3	or CT-positive	2			positive	1				
Heart rate (bpm)	> 120	2	≥ 120	1	> 105	1	≥ 120	1			> 110	1
Systolic blood presure (mmHg)	< 100	4	≤ 90	3	< 110	1	≤ 90	1			< 110	1
	< 120	1										
	< 7	8	≤ 7	10	≤ 11	1			≤ 11	1	< 11	1
	< 9	6	7.1 to 10	1								
Hemoglobin (g/dl)	< 10	4										
	< 11	3										
	< 12	2										
	< -10	4	BD > 5	1							≤ -6	1
Base excess (mmol/L)	< -6	3										
	< -2	1										
Mechanism of injury							penetrating	1	penetrating	1		
INR					> 1.5	1			> 1.5	1		
GCS			≤ 8	1								
Lactate					≥ 5	1						

AIS: abbreviated injury scale; IF: intra-abdominal fluid: FAST: focused assessment with sonography for trauma; INR: international normalized ratio; GCS: Glasgow coma scale; TASH: trauma-associated severe hemorrhage score; Pts: points; BD: Base Deficit; PWH: Prince of Wales Hospital Score; ABC: assessment of blood consumption; CT: computer tomography.

### TASH (trauma-associated severe hemorrhage) score

The TASH score was initially developed and validated on the basis of data from 6,044 blunt-trauma severely injured patients derived from TraumaRegister DGU^®^. Recently, the performance of the TASH score was internally revalidated based on data from 5,834 patients derived from the 2004 to 2007 TraumaRegister DGU^® ^database. TASH uses seven independent, weighted variables to identify patients who will require MT, namely systolic blood pressure, sex, hemoglobin, focused assessment for the sonography of trauma (FAST), heart rate, base excess (BE), and extremity or pelvic fractures. The possible range of scores is between 0 and 28, where each point corresponds to percent risk of MT. An MT was defined by a transfusion requirement of ≥ 10 units of pRBC between ER and ICU admission. The TASH score is transformed into a probability for MT using the following logistic function:

$$\left(\text{p }=\text{ 1}/\left(\text{1}+\text{exp}\left(\text{5}.\text{4}-0.{\text{3}}^{*}\text{TASH}\right)\right)\right).$$

A TASH score ≥ 16 points indicates a probability of MT > 50%. Additionally, the maximum score of ≥ 27 points is associated with a predicted and obtained risk for MT of 100% [22,28].

### PWH (Prince of Wales Hospital/Rainer) score

This model was developed on the basis of a retrospective analysis of 1,891 civilian trauma patients (2001 to 2009) derived from a single center administrative trauma database (PWH Trauma Registry). The Prince of Wales Hospital is a university tertiary referral center located in the New Territories of Hong Kong where 95% of the population are Chinese. Ninety-two patients required ≥ 10 units of pRBC within 24 hours and thus met the criteria for MT. Univariate analysis followed by multivariate stepwise logistic regression identified seven variables to predict the need for MT: heart rate ≥ 120 bpm, systolic blood pressure ≤ 90 mmHg, Glasgow coma scale ≤ 8, displaced pelvic fracture, computer tomography (CT) scan- or FAST-positive for fluid, base deficit > 5 mmol/l, hemoglobin ≤ 7 g/dl, and hemoglobin 7.1-10.0 g/dl [19].

### Vandromme score

The score suggested by Vandromme and colleagues stems from a retrospective analysis of datasets from civilian trauma patients admitted to a single verified level I trauma center in the United States [21]. Based upon three previous studies of MT in the combat setting, clinical characteristics associated with the need for MT were extracted from medical records. MT was defined as the transfusion of 10 units or more of pRBC in the first 24 hours after admission. Clinical measurements used to create the model included: blood lactate (BL) ≥ 5 mmol/l, heart rate > 105 bpm, INR > 1.5, hemoglobin ≤ 11 g/dl, and systolic blood pressure < 110 mmHg [20].

### ABC (assessment of blood consumption) score

The ABC score published in 2009 by Nunez and coworkers [18] was developed based upon a cohort of primary civilian trauma patients. This score uses only non-laboratory and non-weighted parameters that are available during the first minutes after admission of a trauma patient. The parameters include: penetrating mechanism, systolic blood pressure ≤ 90 mmHg on ER arrival, heart rate ≥ 120 bpm on ER arrival, and positive FAST examination. MT was defined as the transfusion of 10 units or more of pRBC in the first 24 hours after admission. Data for score development were collected at the Level I Vanderbilt University Medical Center (VUMC) between 2005 and 2006 and the score was based on data from 596 patients [18].

### Schreiber score

This was one of the first scores predicting massive transfusion in the military setting. Schreiber and colleagues [20] performed a retrospective cohort analysis in two combat support hospitals in Iraq. Included in the study were 558 combat victims, of whom 247 (44.3%) required MT. Variables that independently predict the need for MT were: hemoglobin, INR, and a penetrating mechanism of injury. MT was defined as delivery of ≥ 10 units of a combination of stored RBCs and fresh whole blood (FWB) in the first 24 hours after injury [20].

### Larson score

Another scoring system derived from a combat database is the score reported by Larson and co-workers [29]. These authors performed a retrospective review of the Joint Theater Trauma Registry transfusion database for all US service personnel injured in combat during overseas contingency operations. This score includes: heart rate, systolic blood pressure, hemoglobin and base deficit. MT was defined as 10 units of pRBC within the first 24 hours after admission. Data were collected between 2003 and 2008 from a population of 1,124 patients. There were 420 patients (37%) with MT and 704 (63%) patients without MT [29].

### Statistical analysis

The statistical analysis of this study was based upon the database of the TraumaRegister DGU^® ^(2002 to 2010). Having extracted a dataset of patients with data necessary to reproduce all scores assessed here, the different scores were applied and the area under the receiver operating characteristic curves (AUCs) were calculated, with occurrence of MT as the state variable. From the AUC curves, the cut-off with the best relationship between sensitivity and specificity was used to recalculate sensitivity, specificity, positive predictive value (PPV), and negative predictive value (NPV) for each score, to make scores comparable. The comparison of two AUC curves was based upon the 95% confidence interval (CI) for each curve. From these CIs we derived the standard error (SE) for each curve and applied parametric statistics (*t*-test) to test for differences. A two-sided *P*-value < 0.05 was considered statistically significant. Data on basic characteristics were reported as mean ± SD, and median and interquartile range. All statistical analyses were performed using IBM SPSS 19 (IBM SPSS Inc, Chicago, IL, USA).

## Results

A total of 5,147 (9%) patients were extracted from a complete dataset of 56,573 patients derived from the TraumaRegister DGU^®^. The mean age of patients was 45.7 ± 19.3 years with a mean ISS of 24.3 ± 13.2. The overall MT rate was 5.6% (*n *= 289), and 95% patients (*n *= 4,889) had sustained blunt trauma. The mean time from emergency department (E) admission to ED discharge for the patients analyzed in this cohort was 194.4 ± 146.3 minutes. Table 2 provides an overview of demographics of the study cohort, the baseline physiological data and details of transfused blood products.

**Table 2:** Basic demographic and clinical characteristics.

Clinical Characteristics	**Mean **± **SD**	Median (IQR)	Number (%)	Valid n
Age	45.7 ± 19.3	44.0 (29.0, 60.0)		5147
Sex, male			3780 (73.4%)	5147
ISS	24.3 ± 13.2	22.0 (14.0, 32.0)		5147
NISS	30.4 ± 16.0	27.0 (17.0, 41.0)		5147
Blunt trauma			4889 (95%)	5147
				
Heart rate in ER, bpm	89.7 ± 20	90.0 (78.0, 100.0)		5147
Blood pressure in ER, mmHg	125.3 ± 28.6	125.0 (110.0, 140.0)		5147
Platelet count, *1000/μl	206.6 ± 76.2	203.0 (161.0, 248.0)		5127
Body temperature in ER,°C	36 ± 1.2	36.2 (35.4, 36.9)		3134
				
Hemoglobin in ER, g/dl	11.8 ± 2.6	12.1 (10.1, 13.7)		5147
Base excess in ER, mmol/l	-2.6 ± 4.3	-2.2 (-4.6, 0.0)		5147
Lactate in ER, mmol/l	3.1 ± 5.2	1.9 (1.2, 3.0)		5147
pH in ER	7.3 ± 0.1	7.3 (7.3, 7.4)		2097
aPTT in ER, sec	31.6 ± 12.9	29.0 (25.9, 33.4)		4433
Quick in ER	81.2 ± 21.1	84.0 (69.0, 97.0)		5147
				
pRBC	2.1 ± 5.5	0.0 (0.0, 2.0)		5147
Platelet concentrate	0.1 ± 0.7	0.0 (0.0, 0.0)		4520
Fresh frozen plasma	1.5 ± 4.5	0.0 (0.0, 0.0)		4866
Massive transfusion			289 (5.6%)	5147

IQR represents the 25^th ^to 75^th ^interquartile range. ISS: injury severity score; NISS: new injury severity score; ER: emergency room; aPPT: activated partial thromboplastin time; pRBC: packed red blood cells; ICU: intensive care unit.

**Figure 1:**
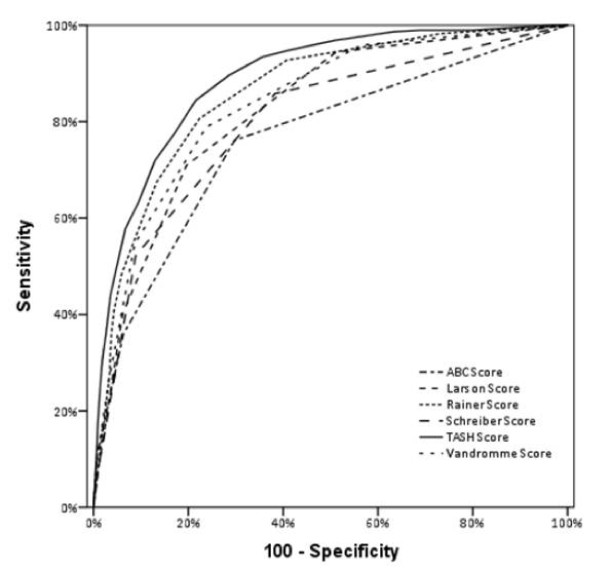
**Validation of six scoring systems and algorithms on one dataset (*n *= 5,147) of severely injured patients extracted from the TraumaRegister DGU^® ^database**. The TASH-Score was internally re-validated while all other scores were externally validated. The two weighted scores (TASH and PWH/Rainer) performed superior over the others.

Figure 1 depicts the AUC for all six scores and algorithms assessed in this study. The TASH score had the highest overall accuracy as reflected by an AUC of 0.889, followed by the PWH score (0.860) and the score developed by Vandromme and colleagues (0.840). The ABC score of Nunez and colleagues performed less accurately than all other scores as reflected by an AUC of 0.763. The performance of each score is summarized in Table 3.

**Table 3:** Performance of compared scores.

	TASH	Rainer	Vandromme	Larson	Schreiber	ABC
AUC	0.889	0.860	0.840	0.823	0.800	0.763
95% CI	0.871, 0.907	0.839, 0.881	0.817, 0.863	0.800, 0.847	0.773, 0.828	0.732, 0.794
Cut-off point	≥ 8.5	≥ 2.5	≥ 1.5	≥ 1.5	≥ 0.5	≥ 0.5
Sensitivity, %	84.4	80.6	78.9	70.9	85.8	76.1
Specificity, %	78.4	77.7	76.2	80.4	61.7	70.3
PPV, %	18.9	17.7	16.5	17.4	11.8	13.2
NPV, %	98.8	98.5	98.4	97.9	98.7	98.0

AUC: area under the curve; CI: confidence interval; PPV: positive predictive value; NPV: negative predictive value; TASH: trauma-associated severe hemorrhage score; ABC: assessment of blood consumption.

Table 3 shows the sensitivity, specificity, positive predictive values (PPV) and negative predictive values (NPV) at defined cut-off values for each score based upon the best relationship between sensitivity and specificity. When considering these cut-offs the highest sensitivity was observed for the Schreiber-Score (85.8%) but also the lowest specificity (61.7%). The TASH score, at the defined cut-off ≥ 8.5, showed a sensitivity of 84.4% and also a high specificity (78.4%). The PWH score had a lower sensitivity (80.6%) with comparable specificity. The Larson score showed the lowest sensitivity (70.9%) at a specificity of 80.4%. These results mirror the results from the AUC calculations in that the TASH score, was generally statistically superior, followed by the PWH scores, with the a statistically significant difference between these two best-performing scores (*P *= 0.0413).

## Discussion

The early aggressive management of the acute coagulopathy of trauma via activation of massive transfusion (MT) protocols including balanced ratios of blood products and the early substitution of coagulation factors may improve survival in the trauma population [7-9]. However, the timely identification of lethal exsanguination remains challenging. This study validated six scoring systems and algorithms to risk-stratify patients for MT at a very early stage after trauma, using a single dataset of severely injured patients derived from the civilian TraumaRegister DGU^® ^database. This dataset included data from primary patients with blunt trauma (95%) of relevant magnitude (ISS > 9) and with the need for intensive care.

Our results show, that the performance of the TASH score was superior with an AUC of 0.889, followed by the PWH score (AUC 0.860). It is unsurprising that the PWH score performed with similar accuracy, as to some extent it incorporates almost identical components and also applies weights to selected individual parameters, albeit following a different pattern to the TASH score. All other scores with fewer parameters and absence of weighting performed less accurately. The score published by Vandromme and colleagues (AUC 0.840) performs almost as well as the PWH score by only using five parameters, from which four are available on clinical investigation (measurement of heart rate and systolic blood pressure) or using point-of-care devices (for measurement of haemoglobin and lactate). It is a not weighted score, which makes it easy to use. The inferior performance of both military scores when applied to our dataset calls into question their applicability for use in the civilian trauma setting, in which the majority of patients suffer blunt trauma. In our dataset the rate of blunt trauma was 95%.

The TASH score has previously been developed and validated in the TraumaRegister DGU^® ^database, and has now been internally revalidated using a different patient cohort. Obviously, this gives the TASH score a somewhat unfair advantage over other scores and may bias the results. In contrast, the other five scores and algorithms have been developed and validated in other databases and were thus externally validated by the present study. The two military scores, Schreiber and Larson scores, that had initially been developed using data derived from combat databases comprising mostly penetrating trauma, were here externally validated on a civilian dataset who mostly suffered blunt trauma. The PWH score, initially developed and validated in the setting of the New Territories of Hong Kong where 95% of the population are Chinese, was validated here on a Caucasian population.

In the past, a few other studies have used a similar approach in externally validating selected scores by their application in other databases, but comparing a maximum of only three scores at a time and including only limited numbers of patients. Here we report the results for six of the most commonly used and cited scores in the literature applied to a total of 5,147 patients from a single trauma database.

Several authors have compared their own scoring system to other existing scores and algorithms [18,30]. For example, Nunez and co-workers have developed and validated their ABC score on their local database and compared its performance to TASH and the Mc-Laughlin score when applied to the same dataset. The differences between the three scores were not statistically significant [18]. In an external validation step, Mitra and colleagues applied three different scores, including the PWH, TASH, and the ABC score, in 1,234 patients derived from The Alfred Trauma Registry, a single center Australian trauma database [17]. The AUC for the PWH score was significantly less than that for the TASH score and was significantly greater than for the ABC score. In another approach, Cotton and co-workers validated the ABC score across multiple demographically diverse level I trauma centers and reported a sensitivity and specificity for the ABC score predicting MT from 75% to 90% and 67% to 88%, respectively [30]. In our study, the sensitivity and the specificity of this score was only 76% and 70%, respectively.

For the present analysis, only scoring systems and algorithms predicting MT with requirement for blood products that could be reproduced by the given TraumaRegister DGU^® ^data extract were considered. Some scores were intentionally excluded from the present analysis due to the data not being captured in the TraumaRegister DGU^® ^to reproduce these scores. These scores and algorithms were, for example, the McLaughlin score, the coagulopathy of severe trauma (COAST) score and the Baker score. McLaughlin published a predictive model for MT based on data from combat casualties [16]. Mitra and co-workers developed the COAST score to accurately identify patients with acute traumatic coagulopathy (ATC) [17], while Baker and colleagues suggested an algorithm to identify risk factors for blood transfusion including four risk factors such as systolic blood pressure, GCS, heart rate and high-risk injury [15]. In the latter case, for example, we were not able to reproduce the high-risk injury based on the data available from the TraumaRegister DGU^®^. The prognostic emergency trauma score (EMTRAS), which has also been developed from data derived from the TraumaRegister DGU^® ^and which may also work for MT, was left out due to mortality as its endpoint [31].

With regard to the clinical applicability of a predictive score in the ED, timely availability of the required parameters is of fundamental importance. Time consuming laboratory analysis or radiographic examination may be good predictors for the need of MT but are not available within appropriate time frames. The TASH score has been proven to be calculated within 10 minutes after the patient's arrival in the ED. This score, however, also relies on laboratory diagnostics for hemoglobin and base excess (BE) but not on global coagulation tests. Most time-consuming assays usually include the assessment of coagulation parameters. Davenport, for example, reported turn-around times for prothrombin time at a median of 78 (62 to 103) minutes [14]. Vice-versa, point-of-care devices as alternatives to conventional laboratory assays, may be inaccurate as point-of-care prothrombin time-ratio has poor agreement with laboratory prothrombin time-ratio in patients with acute traumatic coagulopathy, with 29% false-negative results [14].

In order to quickly assess the coagulation status of a bleeding patient in the ED, rotational thromboelastometry has been advocated to identify acute traumatic coagulopathy at 5 minutes and to predict the need for MT. Davenport and colleagues used rotational thromboelastometry to identify an appropriate diagnostic tool for the early diagnosis of acute traumatic coagulopathy and validated this modality through prediction of transfusion requirements in traumatic hemorrhage. They found that in acute traumatic coagulopathy, the rotational thromboelastometry clot amplitude at 5 minutes was diminished by 42%, and this persisted throughout clot maturation. Finally they pointed out that the clot amplitude at 5 minutes could identify patients who would require MT (detection rate of 71%, vs. 43% for prothrombin time ratio > 1.2, *P *< 0.001) [14]. Schöchl and colleagues used whole-blood thromboelastometry (ROTEM^®^) tests to provide immediate information about the coagulation status of patients with acute traumatic bleeding [32]. They found the best predictive values for MT were provided by hemoglobin and the Quick value (AUC 0.87 for both parameters). However, they also they found similarly high predictive values for FIBTEM MCF (AUC 0.84) and FIBTEM A10 (clot amplitude at 10 minutes, AUC 0.83). They conclude that FIBTEM A10 and FIBTEM MCF provide similar predictive values for MT in trauma patients to the most predictive laboratory parameters [32]. To date, a score using rotational thromboelastometry as a predictor for MT is unavailable, although in some specialist centers thromboelastometry is an instrument that is already used for the prediction of MT in the ED to detect the need for MT and for early application of hemostatic agents.

Cotton and co-workers presented their results of a prospective pilot study (*n *= 272) comparing conventional coagulation testing (CCT) with rapid thrombelastography (r-TEG) showing that early r-TEG values (ACT, k-time, and r-value) were available within 5 minutes, late r-TEG values (maximal amplitude and angle) within 15 minutes, and CCTs within 48 minutes (*P *< 0.001). They conclude that r-TEG results correlate with the CCTs that are not as rapidly available, and are predictive of early transfusions of pRBC, plasma, and platelets [33]. However, this advanced technology may not be available in most trauma centers and therefore easy-to-use and quickly applicable strategies for early assessment and risk stratification, for example via clinical scoring, might be the more logical approach for broad clinical use in daily practice. It is not yet known whether one (ROTEM^®^) is superior over the other (scores/algorithms) and maybe a combination of both is worthwhile until a prospective clinical evaluation has been made.

The present study has certain limitations. A rather substantial number of patients had to be excluded from the analysis due to missing data, and this might have biased the results. Some laboratory parameters, with particular reference to MT (e.g., fibrinogen, protein C) are not documented at all in the TraumaRegister DGU^®^. The data are furthermore limited as the TraumaRegister DGU^® ^has been designed to register severely and/or multiply injured patients requiring ICU admission only. Patients who received hemostatic agents such as fibrinogen, PCC, rFVIIa or any antifibrinolytics with potential influence on the amount of administered pRBCs were excluded, because some of the hemostatic agents (such as fibrinogen and rFVIIa) are not routinely captured in the database. For the present analysis, a single dataset from the TraumaRegister DGU^® ^had to be produced containing all variables for the six scoring systems and algorithms that were assessed. Furthermore, detailed data on transfusion practice and use of blood products had to be available. There may also have been significant bias, as the TASH score was validated on the same database that was used for the initial development. It would be advantageous to validate all scores using a multi-center database, in which none of the scores have previously been developed or validated. Finally, a major and common limitation to all models is related to their retrospective nature and prospective validation of these scores is urgently needed.

## Conclusions

The timely identification of lethal exsanguination remains challenging. This study validated six scoring systems and algorithms to stratify patients at risk for MT at a very early stage after trauma. We found the TASH (AUC 0.889) and the PWH (AUC 0.860) scores perform superiorly to other tested scores. In general, weighted and more sophisticated systems including higher numbers of variables perform better than simple non-weighted models. However, scores for prediction of MT can only guide a clinician in the decision-making process and should not be used alone. Thrombelastography and thromboelastometry devices seem to be promising tools in the treatment and care of patients with severe bleeding. More research is needed to implement the use of these devices as a standard operating procedure.

## Key messages

• The timely identification of lethal exsanguination remains challenging and predictive scores may support early stratification of patients at risk.

• A score needs to be accurate in its prediction as well as rapidly applicable.

• This study validated six scoring systems and algorithms to stratify patients at risk for massive transfusion at a very early stage after trauma, using a single dataset of severely injured patients from the TraumaRegister DGU^® ^database.

• In general, weighted and more sophisticated systems including higher numbers of variables perform better than simple non-weighted models.

• Prospective validations are needed to improve the development process and use of scoring systems in the future.

## Abbreviations

ABC: assessment of blood consumption; AIS: abbreviated injury scale; aPPT: activated partial thromboplastin time; ATC: acute traumatic coagulopathy; AUC: area under the receiver operating characteristic curve; BE: base excess; CCT: conventional coagulation testing; CI: confidence interval; COAST: coagulopathy of severe trauma; CT: computer tomography; ED: emergency department; EMTRAS: emergency trauma score; ER: emergency room; FAST: focused assessment with sonography for trauma; FFP: fresh frozen plasma; FWB: fresh whole blood; GCS: Glasgow coma scale; ICU: intensive care unit; ISS: injury severity score; INR: international normalized ratio; MT: massive transfusion; MTP: massive transfusion protocol; NPV: negative predictive value; pRBC: packed red blood cells; PCC: prothrombin complex concentrate; PPV: positive predictive value; PT: prothrombin time; PWH: Prince of Wales Hospital; rVIIa: recombinant activated factor VII; ROC: receiver operating characteristic; r-TEG: rapid thrombelastography; SE: standard error; TASH: trauma-associated severe hemorrhage score; TR-DGU: TraumaRegister DGU^® ^of the German Society for Trauma Surgery (DGU); TRISS: trauma and injury severity score.

## Competing interests

We report no conflicts of interest. This is an unfunded study.

## Authors' contributions

TB contributed to study design, acquisition of data, interpretation and preparation of the paper. UN and RL contributed to analysis and interpretation of data and revision of the article. MM and SP contributed to study design and revision of the article. AW and BB contributed to study design and revision of the article. MM contributed to study conception and design, acquisition of data, analysis and interpretation of data, and revision of the article. All authors have read and approved the manuscript for publication.
